# Innovative laboratory methods for improved tuberculosis diagnosis and drug-susceptibility testing

**DOI:** 10.3389/ftubr.2023.1295979

**Published:** 2024-01-05

**Authors:** Nathan Mugenyi, Nelson Ssewante, Joseph Baruch Baluku, Felix Bongomin, Mutuku Mukenya Irene, Alfred Andama, Pauline Byakika-Kibwika

**Affiliations:** ^1^Faculty of Medicine, Mbarara University of Science and Technology, Mbarara, Uganda; ^2^Child Health and Development Centre, College of Health Sciences, Makerere University, Kampala, Uganda; ^3^Division of Pulmonology, Kiruddu National Referral Hospital, Kampala, Uganda; ^4^Department of Medical Microbiology and Immunology, Faculty of Medicine, Gulu University, Gulu, Uganda; ^5^Department of Medical Microbiology and Immunology, School of Medicine, Kabale University, Kabale, Uganda; ^6^Department of Medicine, Makerere University College of Health Sciences, Makerere University, Kampala, Uganda

**Keywords:** innovative laboratory methods, TB diagnosis, drug susceptibility testing, treatment monitoring, diagnostic methods - policies

## Abstract

The laboratory plays a vital role in the diagnosis of all clinical forms of tuberculosis (TB), from microbiological confirmation of *Mycobacterium tuberculosis* to drug-susceptibility testing (DST) and treatment monitoring. For many decades, laboratory diagnosis of tuberculosis was based on conventional methods such as smear microscopy, and culture-based methods. However, *Mycobacterium tuberculosis* is a slow-growing organism, requiring 2–4 weeks or longer for cultures to yield results. Therefore, the evaluation of novel and rapid diagnostic methods has been a priority for research and development. In the beginning of 1990s, molecular-based diagnostics became widely available providing rapid detection, identification, and DST of *M. tuberculosis*. In this paper, we review some of the new diagnostic methods introduced in the clinical laboratory for the diagnosis of tuberculosis. With the global goal of ending TB as a public health challenge by 2030, enhancing diagnostic capabilities for latent and active TB, along with improving DST, would improve identification and management of cases, reducing transmission rates and curbing the spread of drug-resistant strains. These innovations promise to transform TB control efforts, bringing us closer to eradicating this persistent global health threat.

## Introduction

Tuberculosis (TB) is the leading infectious cause of death globally, and is responsible for more than 1.6 million deaths and about 10.6 million new cases annually (1). TB is caused by the *Mycobacterium tuberculosis* complex consisting of *M. tuberculosis, M*. *africanum, M. bovis, M. canettii, M. microti, M. pinnipedii, M. orygis* and *M. caprae* (2). As primarily a lung disease, TB is acquired through inhalation of droplet nuclei containing the causative agent (3). Extra-pulmonary spread and multi-organ involvement or disseminated disease occurs, particularly in immuno-compromised hosts (4). Early detection and treatment of active and latent TB are key factors in the control of the TB epidemic. Globally, it has been estimated that only 64% of TB cases are detected, indicating that 36% of the cases are not identified (5).

Confirmation of pulmonary TB infection should be performed in the laboratory using respiratory samples such as sputum (spontaneously expectorated or induced), broncho-alveolar lavage, nasal washings, or sampling gastric contents) (6).

Enhancing laboratory capacity and performance is crucial for TB control since laboratories play a pivotal role in the diagnosis of TB (7). Smear microscopy along with other tests are used for the diagnosis of TB in over 70% of laboratories in low and middle-income countries (LMICs), but smear microscopy has low sensitivity and can detect only about 60% of TB cases (8). In developed countries, the choice of diagnostic tests is guided by the need for accuracy and speed, as well as the clinical presentation of the patient. Smear microscopy, with its lower sensitivity, is not the primary method of TB diagnosis. It can still be used in some cases, such as for initial screening in resource-constrained settings, but the emphasis is on complementing it with more sensitive and specific tests like culture and molecular assays to ensure accurate and timely diagnosis. This approach is essential for effective TB management, reducing transmission, and addressing drug-resistant TB cases.

Additionally, about 25% of all TB cases involve infections outside of the lungs, and these presentations are often not diagnosed with smear microscopy (8). Culture is able to identify cases with low levels of *Mycobacterium* and provides additional benefits such as DST and identification of other *Mycobacterium* species (8). Due to the slow growth of *Mycobacteria*, results take 3–4 weeks or longer on Lowenstein Jensen (LJ) medium and about 2 weeks in liquid *Mycobacteria* Growth Indicator Tubes (MGIT)-960 medium, therefore faster and more accurate diagnostic tests are required to improve patient management (9).

Cutting-edge technologies in laboratory medicine have been developed for early detection of *Mycobacterium* and assessing drug susceptibility. These utilize liquid culture medium, nucleic acid amplification techniques (NAATs), DNA hybridization, mutation detection techniques, antibody, and antigen detection (10). This review discusses new laboratory techniques currently available for the diagnosis of TB and DST.

## Advances in the diagnosis of pediatric tuberculosis

Approximately 1.2 million children were among the 10.6 million people worldwide who became sick with TB in 2021. Diagnosing and treating TB in children is challenging despite the fact that it is a preventable and treatable disease (11). Traditional samples such as sputum and gastric aspirates for TB testing are not very useful for diagnosing TB in infants as they are difficult to collect, making diagnosis of pediatric TB a significant challenge (11). The ease of stool collection from individuals makes it a significant advancement in the new technologies of TB diagnosis as it can be utilized for TB testing (11). The diagnosis of childhood TB has been significantly improved by an innovative stool sample processing kit (12). The development of the stool sample processing kit aimed to investigate the feasibility of utilizing an automated PCR test with stool samples as an alternative to culture techniques and smear microscopy in the diagnosis of TB in children (13). The kit is designed to process stool samples without requiring any specialized laboratory equipment or skilled personnel, and it works together with the Cepheid Expert MTB/RIF Ultra assay (14). Stool-based diagnostic tests have a sensitivity of about 60% in children with confirmed TB, but their sensitivity is very low (2–6%) in clinically diagnosed TB (14).

## Improvement in smear microscopy for TB diagnosis

Microscopic examination of Ziehl-Neelsen (ZN) stained sputum smears has been in use for nearly 100 years (15). The light microscope smear microscopy method is not very effective for diagnosing TB, as it has low sensitivity (16), and it only has the ability to detect about 60–70% of TB cases (16). An alternative to light microscopy for TB diagnosis is fluorescence microscopy, which is reported to be more sensitive by 10% (17, 18). This is because the fluorescent bacilli of *M. tuberculosis* can be observed at a lower magnification and a larger field of view than with light microscopy (17). On the other hand, fluorescence microscopy has some drawbacks compared to light microscopy. It requires a microscope with a mercury vapor lamp, which is more expensive, and the ultra violet lamp needs to be replaced frequently, about every 200–300 h (19). Additionally, the slides need to be read in a dark room, which can be an inconvenience (18).

Light Emitting Diode (LED) fluorescence microscopy is a recent technological improvement that uses an illumination system based on a light-emitting diode that has a much longer lifespan of 10,000 h compared to the traditional fluorescence microscope which uses a mercury vapor lamp (20). LED-based fluorescent microscopes are now accessible and reasonably priced (21). The effectiveness of these microscopes was evaluated by the WHO, and the findings indicated that their diagnostic accuracy is higher by approximately 10% compared to traditional smear microscopy (21).

## Alternative culture-based methods for TB diagnosis

Isolation of *M. tuberculosis* on a culture medium has for a long time been regarded as the gold standard for the diagnosis of TB (22). Culture techniques also allow for the identification of specific isolates through either biochemical tests or molecular methods and DST (23). Solid egg-based media such as LJ and Stonebrink medium were commonly used for culture of TB until the early nineties (24).

Stonebrink medium is a specialized culture medium used for the isolation and growth of *M. bovis*. The LJ base contains ingredients such as agar, glycerol, malachite green and other nutrients. Alternatively, it contains bromothymol blue, a pH indicator that detects the production of acid by *M. bovis* colonies. The medium changes color as *M. bovis* metabolizes specific substrates. Stonebrink medium is primarily used in veterinary diagnostic laboratories and research settings to isolate and identify *M. bovis* from clinical specimens, particularly from bovine sources (25). However, a disadvantage of these solid media is the slow growth of the bacteria, which can take up to 8 weeks before the culture results are confirmed (24). The sensitivity of liquid culture is higher by up to 20% and the time required for detection is shorter (10–14 days compared to 2–4 weeks) than traditional solid media (26). Therefore, WHO recommends using both traditional solid media and liquid media for the primary isolation of *Mycobacteria* (26). To improve the accuracy of TB diagnosis, a liquid broth culture can be used. In this culture, Middlebrook 7H9 broth, which is supplemented with 10% OADC (oleic acid, albumin, dextrose, and catalase) and PANTA (an antibiotic mixture of polymyxin, amphotericin B, nalidixic acid, trimethoprim, and azlocillin) to prevent contamination from other microorganisms are added (27). Various commercial culture systems are currently available, including simple bottles and tubes such as MGIT (BD BACTEC^TM^ MGIT^TM^), Septi-Chek Acid Fast Bacilli (BBL-Becton Dikinson Microbiology Systems), and MB Redox, as well as more advanced systems such as the semi-automated BACTEC 460TB and fully automated systems like BACTEC 9000 MB and BACTEC MGIT 960 (all from BD, USA), ESP Culture System II (Trek Diagnostics, USA), and MB/BacT ALERT 3D System (BioMérieux, NC) (28).

## Lateral flow lipoarabinomannan

A quick and timely diagnosis is crucial in managing diseases, and point-of-care testing can provide immediate results. A test for TB that can be performed at the point-of-care detects a substance called lipoarabinomannan in urine. LAM is a component of the cell wall of *Mycobacteria*, which is released when cells break down and is excreted in urine after being processed by the kidneys (29). It is a potential biomarker of diagnosis of *M. tuberculosis*. Various brands of a test that detects LAM are available, such as Abbott Determine™ TB LAM Ag in the USA, which was approved by WHO in 2015. Another brand is the Fujifilm SILVAMP TB-LAM test, also known as the FujiLAM, which is a new type of lateral flow LAM test that has a higher sensitivity compared to the Alere Determine™ TB LAM Ag (30). The Urinary LAM is used for the diagnosis of TB among individuals co-infected with HIV and with lower CD4 counts and has demonstrated improved sensitivity. The test was however not been approved for use by WHO committee in 2019 due to its low sensitivity (31) but is currently a good contender as a point-of-care test (30).

Alere Determine™ TB LAM and Fujifilm SILVAMP TB-LAM tests are based on the detection of LAM in urine, there are differences in terms of manufacturer, reported sensitivity and specificity, ease of use, and the potential use as a stand-alone test (32). These differences may influence the choice of test in specific clinical and resource settings. It is therefore essential to consider the patient's clinical condition and the available diagnostic options when deciding which test to use for TB diagnosis.

## Sero-diagnosis of TB

Serology involves detecting antibodies against *Mtb* in serum, which can provide quick and cost-effective results (33). Recent meta-analyses and systematic reviews have determined that the currently available commercial serological tests produce inconsistent results due to issues such as cross-reactivity and poor sensitivity (23). Results from studies showed that sensitivity and specificity of serological tests for both pulmonary and extra-pulmonary TB were inconsistent and imprecise (33). The included studies for this analysis utilized a cohort or case series type design, with culture and clinical diagnosis being used as reference standards. In terms of pulmonary TB (8 test evaluations), commercial serological tests demonstrated modest performance with a pooled sensitivity of 88 percent and a pooled specificity of 50 percent, resulting in a diagnostic odds ratio (DOR) of 7.30 (95% CI 1.95, 27.24). However, when only studies meeting at least two methodological quality criteria were considered (representative patient population and blinding of the serological test result), the DOR decreased to 6.35 (95% CI 0.59, 67.98) and the sensitivity decreased to 34 percent. For extra-pulmonary TB (4 test evaluations), the pooled sensitivity was <50% while the pooled specificity was 93 percent (34). The WHO advises against the use of commercial serological tests for diagnosing pulmonary and extra-pulmonary TB (35).

Antigen detection has gained attention as a promising approach for TB diagnosis. Immuno-PCR (iPCR) and real-time immuno-PCR are techniques that combine immunoassay principles with the amplification power of PCR. Immuno-PCR (I-PCR) integrates the ease and adaptability of ELISA with the substantial amplification capability and heightened sensitivity of PCR, resulting in a notable enhancement in sensitivity when compared to a similar ELISA (36). These methods can enhance the sensitivity and specificity of detection (37). Recent research in this field has focused on identifying and validating TB-specific antigens for use in immuno-PCR. These assays aim to improve the accuracy of TB diagnosis, especially in cases of extrapulmonary TB where bacillary load is low (38).

Aptamers are single-stranded DNA or RNA molecules that can bind specifically to target molecules, including proteins and antigens. Aptamer-based assays have been explored for their potential in TB diagnosis (39). Aptamers can be designed to bind to TB-specific antigens and used in diagnostic tests. These assays offer the advantage of high specificity and sensitivity. The development of aptamer-based assays is an exciting area of research that may lead to more accurate and reliable TB diagnostics. More studies are required to develop immune response-based or serological tests that can provide accurate results (39).

## Diagnosis of latent *M. tuberculosis* infection

Individuals with latent TB infection have been infected with *M. tuberculosis*, but they do not exhibit symptoms of active TB disease (40). Until recently, the only way to detect *M. tuberculosis* infection was through the tuberculin skin test (TST), also known as the *Mantoux* test (41). TST involves intradermally injecting a small amount of a purified protein derivative (PPD) into the skin and waiting for an induration to develop at the injection site within 2 days, indicating exposure to *M. tuberculosis* (41). However, the test cannot distinguish between latent infection and active disease and its limitations have been widely publicized (42). TST can produce inaccurate results due to false positives, which may occur as a result of exposure to non-tuberculous *Mycobacteria* or the Bacillus Calmette-Guerin (BCG) vaccine (43). This is because the PPD used in the test contains similar antigens to those present in BCG and certain non-tuberculous *Mycobacteria*. Despite its limitations, skin testing is still the most commonly used approach for detecting TB infection (44). IFN-γ is an important factor in regulating cell-mediated immune responses against *M. tuberculosis* (45). This understanding has led to the development of alternative tests for detecting TB infection, known as IFN-γ-release assays (IGRAs). These are blood tests that measure the release of interferon (IFN)-gamma from T cells after they have been stimulated with TB-specific antigens (45). Two commercially available IGRAs are Quantiferon TB Gold and T-SPOT TB (45). However, since there is no standard reference test for detecting latent TB infections, it is difficult to determine the accuracy of these assays (45). The occurrence of false negative results in tests for latent TB infection can be attributed to various factors, including simultaneous anergy, recent TB infection, the presence of concurrent viral, bacterial or fungal infections, recent vaccination with live viruses, chronic renal failure, low protein states, diseases that affect lymphoid organs such as Hodgkin's disease, lymphoma, chronic leukemia, and sarcoidosis, immunosuppressive drugs, very young age (<6 months) or elderly individuals, stress, incorrect antigen storage or handling, incorrect administration, and misinterpretation of test results (46). It is important to note that IGRAs are not capable of differentiating between latent TB infection and active TB disease, and therefore should not be used as a diagnostic tool for active TB.

## DNA-based tools for the diagnosis of TB

Numerous techniques have been documented for detecting *M. tuberculosis* using a PCR test. This involves using short sequences of DNA, known as oligonucleotide primers, to amplify a specific DNA fragment that is unique to this microorganism (47). The classic Nucleic Acid Amplification Tests can give results in 3–6 h (47). There are different types of tests available for detecting *Mtb* using PCR, including commercial kits and “in-house” tests (47, 48). The in-house tests are based on a protocol that has been developed in a non-commercial laboratory (47). Each nucleic acid amplification test (NAAT) employs a distinct technique to amplify specific regions of nucleic acid in the genome of the *M. tuberculosis* complex (Table 1). There are various commercial NAATs available, and the U.S. Food and Drug Administration (FDA) has authorized the use of certain commercial NAATs for respiratory specimens exclusively (47). Several kits, including the Gene-Probe Amplified *M. tuberculosis* direct test (AMTD), the Roche Amplicor MTB test, the Cobas Amplicor test, the Abbott LCx test, and the BD-ProbeTec (SDA) test, are available for detecting *M. tuberculosis* (47). However, none of these methods have been authorized for direct detection of *M. tuberculosis* from extra-pulmonary specimens. While these technologies are rapid and highly specific, their performance characteristics may vary, and their sensitivity is still not as good as that of culture-based methods, especially for samples that are negative on smear microscopy (47).

**Table 1:** Summary of some of the TB diagnostic tests.

**Test**	**Principle**	**Technique**	**Diagnostic Performance**	**Sensitivity**	**Advantages**	**Disadvantages**
**Microscopy sputum smear**	Microscopic examination of specially stained smears to detect acid-fast organisms such as *Mycobacterium tuberculosis* and non-tuberculous *mycobacteria*. Acid fastness is a physical property that gives a bacterium the ability to resist decolorization by acids during staining procedures.			• 50–60% HIV negative patients • <30 % in People Living with HIV (PLWHIV) and Children (18).	• Simple, Fast, inexpensive, and rapid • Vital tool in Laboratory TB diagnosis in low and middle income countries. • Suitable for low-level and high-level laboratories, including laboratories with low biosafety levels. • Can be used for treatment monitoring • Widely available	• Needs a large number of bacilli per ml of specimen to be detected positive. • Can only be used for pulmonary TB • Cannot indicate for drug susceptibility or resistance. • Relies only on sputum
	Primary stain Auramine (fluorochrome dye) that forms a complex with Mycolic acid of the cell wall. Decolorization (strong acids, alcohol) does not release primary stain from the cell wall.	**A. Light-emitting diode fluorescence microscopy (LED-FM)** • Auramine staining / Fluorochrome staining.	• The *mycobacteria* visualized under ultraviolet light retain the fluorescent bright yellow color of Auramine.			• Higher cost associated with purchase of the microscope with a mercury vapor lamp and the need for frequent replacement of the Ultra violet lamp. • Cannot differentiate between *M. tuberculosis* and other Acid Fast bacteria.
	The lipoid capsule of the acid-fast organism takes up carbol-fuchsin and resists decolorization with a dilute acid rinse.	**B. Bright field microscopy** • Ziehl-Neelsen/ Carbol-fuchsin staining contrast red AFB on • Blue green background (Methylene blue) • Green background (Malachite green)	• The acid-fast bacilli will stain bright red, and the background will stain blue.			
**Culture**	Culture test involves studying bacteria by growing the bacteria on different substances. This is to find out if particular bacteria are present. In the case of the TB culture test the test is to see if the TB bacteria *Mycobacterium* tuberculosis, are present.	**Solid culture**	• A selective egg-based medium used to culture and isolates Mycobacterium species, including *Mycobacterium tuberculosis*, from clinical specimens. • It contains Congo red and malachite green to inhibit unwanted bacteria. • Requires 7–21 days to grow. • Lowenstein Jensen and Middlebrook 7H11S (20).	70–97% Sensitivity (20).	• Colony morphology can be visualized to assist with identification. Gives definite diagnosis of TB. Detects 30%−50% more cases than microscopy (high sensitivity) • Provides the needed isolates for drug susceptibility testing (DST). • Necessary for monitoring DR-TB treatment. • Less overgrowth due to contamination.	• Results take a long time (about two months) due to slow growth. • High biosafety level requirements. • Highly trained staff needed.
		**Liquid culture**	• *Mycobacteria* Growth Indicator Tube (MGIT) system) (49)•BACTEC 960/ MGIT BACTEC 460		• Can process higher number of samples than solid culture (10% higher) • Faster than solid culture (26).	
**Drug sensitivity testing (DST)**	These testing methods are based on the reduction of a colored indicator added to liquid culture medium in a micro-titer plate after *M. tuberculosis* has been pre-incubated for several days *in vitro* to different antibiotics and different drug concentrations.	Culture	Resistance is detected by a change in color of the indicator, which is proportional to the number of viable *Mycobacteria* in the medium. Detection of the colored reaction on the drug-free medium indicates a positive culture and a colored reaction in both drug-free and drug-containing media indicates resistance (22).	70–97% Sensitivity (20).	• DST provides a definitive diagnosis of drug- resistant TB.	• Requires 4 – 8 weeks to produce results. • Non-molecular DST methods take longer to • Provide results • Liquid DST fails to detect some clinically • Relevant “borderline rifampicin resistant strains” with *rpo*B mutations (23).
**Molecular drug resistance testing methods**	It uses a hemi-nested PCR to amplify the rifampin resistance-determining region (RRDR) of the *M. tuberculosis rpo*B gene (8).	**GeneXpert MTB/RIF**^®^ **(Cepheid, Sunnyvale, USA)** Cartridge based nucleic acid amplification test, automated diagnostic test, **GeneXpert MTB/RIF Ultra**^®^ **assay (Ultra) (Cepheid, Sunnyvale, USA)** Cartridge based nucleic acid amplification test, automated diagnostic test,	• Identifies MTB DNA and Resistance to Rifampicin (RIF) by Nucleic Acid Amplification Test) • Detection of a single copy target: *rpo*B gene (5 probes) • Detection of a single copy target: *rpo*B gene (4 probes). • Detection of 2 different multi-copy targets: IS6110 and IS1081 (2 probes)	• Sensitivity 88% for Smear positive • 68% sensitivity for smear-negative culture-positive TB • Rifampicin resistance, Xpert MTB/ • RIF has a sensitivity of 95% and specificity of 98% (24). • 85% sensitivity for smear-negative culture-positive TB	• DST provides a definitive diagnosis of drug- resistant TB. • The five *rpo*B RRDR detect the presence of mutations responsible for 95% of rifampicin resistance and *M. tuberculosis*. • Shorter turnaround time, this assay takes around 110 minutes. • Better performance of Xpert MTB/RIF in detecting TB over that of microscopy (9) (10). • 5% higher compared to the sensitivity of Xpert MTB/RIF (12). • Better discrimination of *rpo*B mutations Silent Mutation reported as susceptible • Detection of RIF resistance in mixed infections (27).	• False identification of RIF-R occasionally occurs and decreased sensitivity in smear-negative sputum (8). • A stable uninterruptable electrical supply is needed. • The shelf life of the cartridges is only 18 months; • A very stable electricity supply is required; • The instrument needs to be recalibrated annually • The cost of the test; • The temperature ceiling is critical (8).
	**Line probe assays: uses PCR and reverse hybridization methods for rapid detection of mutations associated with drug resistance**. The LPA is designed to identify *Mycobacterium tuberculosis* complex (MTBC) and also detect drug resistance mutations.	• GenoTypeMTBDRsl V1 and V2 (Hain Life-science GmbH, Germany) • Nipro (Tokyo, Japan) non-tuberculous *mycobacteria* +MTBDR detection kit 2 • Genoscholar™ NTM+MDRTB II (50)•Genoscholar PZA TB II • Genoscholar FQ+KM-TB II	• Detect fluoroquinolone and amikacin resistance. • Resistance to rifampicin and isoniazid. • Rifampicin and isoniazid. • Resistance to pyrazinamide (5) (16). • Resistance to fluoroquinolone and kanamycin.	Sensitivities: Active TB 68.4 % 2.3% for Extra Pulmonary TB (28).	• Molecular LPAs enable rapid detection (<48 h) • High throughput technology, allowing up to 48 specimens to be processed simultaneously (19).	
**Serological technique**	Detection of antibodies against *M. tuberculosis* in serum.	• Using the enzyme-linked immunosorbent assay (ELISA) • Using an immunochromatographic assay format.		• Pulmonary TB (28). • Sensitivity : 63–85% • Specificity: 73–100% for pulmonary TB (51).	• It can offer low-cost and rapid results. • Good if sputum sample is difficult to obtain or patient has extra-pulmonary TB (51).	• Sero-diagnosis tests (such as the Anda-TB IgG) have very variable results and this generally means low sensitivity and specificity leading to significant number of “false negative” results.
**Latent TB infection**	Indirectly measure TB infection by detecting memory T-cell response signifying the presence of host sensitization to *M. tuberculosis* antigens	• QuantiFERO-TB • Gold Plus (QFT-Plus) • WANTAI TB-IGRA • SPOT^®^TBLISPOT-based • IGRA(ELISPOT base	• Interferon-gamma release assays • IGRAs are *in vitro* blood tests that measure interferon-gamma released by circulating lymphocytes in whole blood during overnight incubation with exposure to *M. tuberculosis*- specific antigens (ELISA based) or the number of T-lymphocytes producing interferon-gamma	• Sensitivity 65–81% (52).	• Shorter turnaround time 24-48 h • Requires one visit from patient • Does not result into false positives due to high specificity in BCG vaccinated populations (21).	• Expensive • Blood samples need to be quickly processed (<30 h) • Adequately trained staff (21).
**The tuberculin skin test(TST)/ Mantoux test**	• The TB skin test is based on the logic that the infection with *M. tuberculosis* bacterium results in a delayed-type hypersensitivity skin reaction to some components of the bacterium. • The Purified Protein Derivative (PPD) material is administered to the skin for testing tuberculosis infection. • The raised area on the skin site where the PPD was injected indicates the immune cells or T-cells have reacted and attracted by the immune system to the site where the tuberculin protein derivative was released.	Positive result detected by skin reaction (induration) at the point of injection.	• The immune cells release lymphokines at this site which causes hardening of the injection site due to local vasodilatation.	• Sensitivity: 59–99%	• The Mantoux test, a tuberculin skin test, provides a benchmark for TB prevention.	• The test does not differentiate between latent infection and active disease. • False-positive TSTs can result from contact with non-tuberculous *mycobacteria* or vaccination with Bacille Calmette-Guerin (BCG), because PPD, a crude protein preparation, contains antigens that are also present in BCG and certain non-tuberculous *mycobacteria*. • Has relatively low specificity in those with recent BCG vaccination and immune-suppressed individuals (for example PLHIV), • Requires two clinic visits. • Only valid if the results are read within the suggested time frame (WHO consolidated guidelines on tuberculosis).

In a recent meta-analysis that evaluated more than 125 studies on commercial NAATs and their accuracy in detecting *M. tuberculosis* in smear-positive samples, a wide range of accuracy levels was found across the studies. Another meta-analysis that investigated the diagnostic accuracy of nucleic acid amplification tests for pulmonary TB using urine samples revealed that PCR testing for *M. tuberculosis* in urine had high specificity but only modest sensitivity (55%) in diagnosing active pulmonary TB (53). This analysis concludes that there is a need for improvement in the diagnostic accuracy of NAATs, particularly sensitivity and commercial NAATs alone cannot be recommended to replace culture and microscopy for diagnosing pulmonary TB.

Loop-Mediated Isothermal Amplification (LAMP) is a molecular diagnostic technique. This method is based on isothermal amplification of DNA. Since its introduction, LAMP has become a valuable tool for TB diagnosis and has been endorsed by the WHO (54). The LAMP method is founded on the innovative LAMP platform, which was created by Eiken Chemical Company in Japan (55). This technology amplifies the target DNA at a constant temperature of around 65°C and has been designed to directly detect DNA from clinical samples in <2 h, with minimal instrumentation, and using a visual detection system (55). Its high sensitivity, speed, and adaptability to various clinical specimens have made it a valuable tool in improving the accuracy and timeliness of TB diagnosis.

## Drug susceptibility testing

The occurrence and proliferation of multidrug-resistant tuberculosis (MDR-TB) and extensively drug-resistant tuberculosis (XDR-TB) are significant concerns in the medical and public health fields (56, 57).

MDR-TB refers to tuberculosis that has developed resistance to at least two of the first line anti-TB medications, rifampicin and isoniazid (58). Extensively drug-resistant tuberculosis (XDR-TB) is a form of TB that is classified as both MDR/RR-TB and also exhibits resistance to at least one fluoroquinolone drug, as well as one or more Group A drugs. Group A drugs refer to a potent set of second-line medications used in the treatment of drug-resistant TB, including levofloxacin, moxifloxacin, bedaquiline, and linezolid (1).

Detecting drug resistance at an earlier stage is crucial as it shortens the period between the diagnosis of TB and the commencement of treatment, ultimately leading to better outcomes for patients and contributing to the control of resistant strains in the population by reducing their transmission (59). Conventional techniques for testing drug susceptibility are time-consuming (27). The standard proportion method, which is the most widely used approach and is carried out on Lowenstein-Jensen medium or Middlebrook agar, takes approximately 4 to 8 weeks to generate results. This method is considered an “indirect method” as it necessitates a sequential process that involves isolating *Mycobacteria* from the clinical specimen, identifying the *M. tuberculosis* complex, and then performing *in vitro* testing of the strain's susceptibility to anti-TB medications (27).

Over the past 15 years, a range of culture- and molecular-based techniques have emerged, some of which are considered “direct methods” that use patient specimens and avoid the need to first isolate *M. tuberculosis* in a pure culture from clinical samples (60). Several low-cost methods have been suggested for the swift identification of drug resistance, particularly for use in low-income settings. These methods include the microscopic observation of drug susceptibility (MODS), thin layer agar (TLA), colorimetric redox indicator (CRI) techniques, and the NRA (61). These techniques are capable of providing susceptibility results within 1–2 weeks after inoculation. Both MODS and TLA testing involve directly inoculating drug-free and drug-containing media (liquid medium for MODS and solid medium for TLA with patient specimens (61). In these methods, the isolation of pure culture from clinical specimens is not required. Instead, cultures are examined under a microscope for early growth or micro-colonies. If growth is observed in drug-free media, it indicates a positive culture, while growth in both drug-free and drug-containing media suggests resistance. Colorimetric redox indicator (CRI) methods are considered indirect and therefore require a pure culture to be isolated from clinical specimens (37). These methods involve adding a colored indicator to a liquid culture medium in a microtiter plate after *M. tuberculosis* has been pre-incubated for several days with different antibiotics and concentrations of drugs *in vitro*. The color of the indicator is reduced based on the presence or absence of growth, and the degree of color change indicates the level of drug resistance (51). Detection of resistance using colorimetric redox indicator (CRI) methods is based on the change in the color of the indicator which is related to the number of viable *Mycobacteria* present in the medium after incubation with different antibiotics and drug concentration (62). Various growth indicators are utilized in these methods, including Alamar blue and Resazurin. The nitrate reductase assay, on the other hand, is a solid culture approach that utilizes LJ medium (63). It works by detecting the nitrite that is produced when *M. tuberculosis* reduces nitrate. A specific reagent called Griess reagent is added to the medium, which contains 1 mg/ml of potassium nitrate (KNO_3_). The NRA test can be used as a direct or indirect test. To perform resistance testing using the nitrate reductase assay, patient samples or a pure culture of *M. tuberculosis* is directly inoculated onto media containing antibiotics and media without antibiotics. The nitrate reductase assay detects the reduction of nitrate, which is indicated by a colored reaction (63).

The BACTEC MGIT 960 system is based on detecting reduced levels of oxygen in broth as a result of bacterial respiration (64). It is a commonly used automated liquid culture DST system for testing the susceptibility of first-line antibiotics and it is an indirect method which requires a positive culture of *M. tuberculosis* complex with inoculation into a liquid medium consisting of drugs or not (63). It can report susceptibility results in 1–2 weeks after inoculation and has been demonstrated to be equivalent to the standard proportion method. The method has been approved by the FDA and endorsed by the WHO (65).

The BACTEC MGIT 960 system is highly sensitive and specific to detect *Mycobacteria* including drug resistant strains (64). Additionally, it is rapid and can process multiple samples simultaneously. However, the BACTEC MGIT 960 system is relatively expensive to purchase and maintain (64).

## Molecular drug resistance testing

There are important differences in the intended use of molecular drug resistance testing methods such as GeneXpert and Line probe assays (LPAs). GeneXpert is primarily intended to be used on specimens such as sputum or other respiratory samples while LPAs are generally designed for use on cultured isolates of *Mycobacterium tuberculosis* (Table 1).

## Gene Xpert MTB/RIF^®^, Xpert MTB/RIF ultra

Improved technology has led to advancements in the detection of drug resistance to *M. tuberculosis*. The Gene Xpert MTB/RIF^®^ technology from Cepheid, based in Sunnyvale, USA, was endorsed by the WHO in 2010. Since then, it has been implemented on a large scale worldwide (66, 67). The Gene Xpert MTB/RIF^®^ is a type of test that can quickly detect both M. tuberculosis and rifampin resistance and is designed to be used in a point-of-care setting. The Gene Xpert MTB/RIF^®^ utilizes a type of polymerase chain reaction (PCR) called Hemi-nested PCR to amplify the region of the *rpo*B gene in *M. tuberculosis* that determines resistance to rifampin (68, 69). This method can detect mutations in the rifampin resistance-determining region (RRDR) of the gene, which account for 95% of rifampicin resistance cases in *M. tuberculosis* (70, 71). The Gene Xpert MTB/RIF^®^ test, while being useful in detecting *M. tuberculosis* and rifampin resistance, does have some drawbacks. False identification of rifampin resistance can occur on occasion, and the sensitivity of the test can decrease when used on smear-negative sputum (68). The Gene Xpert MTB/RIF Ultra^®^ assay (Ultra) was developed to address limitations of the Gene Xpert MTB/RIF^®^ test (72). A comparison analysis revealed that the sensitivity of the Xpert MTB/RIF Ultra was 5% higher compared to the sensitivity of Xpert MTB/RIF (73).

GeneXpert Ultra and GeneXpert offer significant advantages in TB diagnosis. GeneXpert Ultra is known for its speed and simultaneous detection of TB and rifampicin resistance, making it a valuable tool for rapid diagnosis in a variety of settings (74). GeneXpert excels in terms of sensitivity, allowing it to detect TB in cases with lower bacterial loads and to identify isoniazid resistance. The choice between the two tests depends on the specific clinical context and the desired diagnostic outcomes, with GeneXpert Ultra being particularly beneficial in challenging cases and in settings where resources allow for its use (75).

## Next generation sequencing technique for DST

NGS has emerged as a powerful tool for the detection of drug resistance in *Mtb* isolates however much it is expensive. NGS-based methods for DST offer several advantages over traditional phenotypic DST, including higher sensitivity and the ability to detect multiple resistance mutations simultaneously (76). WHO's End TB strategy has prioritized universal access to early diagnosis and comprehensive DST for all individuals with TB as a key component of integrated, patient-centered TB care (56).

Whole-genome sequencing (WGS) is one of the approaches to NGS-based DST. WGS involves sequencing the entire genome of *Mycobacterium* isolate, allowing for the identification of all genetic variants. Sputum-based WGS technology has advanced in recent years, making it possible to sequence *Mycobacterium* directly from sputum samples, without the need for culture (77). This technology can provide faster turnaround times for results, allowing for more timely and targeted treatment decisions. Targeted sequencing is another approach to NGS-based DST, focusing on specific genes or regions of genome known to be associated with drug resistance (78). This approach can be more efficient than WGS and provides faster results, making it a promising option for clinical use. While NGS-based DST holds great promise for improving TB care, there are still challenges to be addressed. The analysis and interpretation of NGS data is complex and requires specialized expertise and software. In addition, the clinical utility of NGS-based DST is limited by lack of standardized methods and the need for genotypic and phenotypic techniques monitor treatment response to curb the spread of drug resistance (76).

## Line probe assays

LPA is a rapid detection method that uses PCR and reverse hybridization techniques to identify mutations associated with drug resistance in *Mycobacterium tuberculosis* (79). There are different types of LPA tests available, such as the GenoType MTBDRplus version 1, introduced in 2008, the GenoType MTBDRplus version 2 (Hain Life-science GmbH, Germany), introduced in 2011, and the Nipro (Tokyo, Japan) non-tuberculous *mycobacteria* +MTBDR detection kit 2, also introduced in 2011 (80). In 2016, the WHO recommended these LPA tests as the initial method for detecting resistance to rifampicin and isoniazid instead of the phenotypic DST (81). Hain Life science in Germany developed GenoType MTBDRsl (V1 and V2) for testing resistance to second-line TB drugs, specifically fluoroquinolones and amikacin. These tests have been recommended by WHO for use in patients with rifampicin-resistant multidrug-resistant tuberculosis (MDR-TB) to guide appropriate MDR-TB treatment plans (80).

The Genoscholar™ NTM+MDRTB II test from Nipro (Tokyo, Japan) has been approved by the WHO for detecting Mycobacteria species and resistance to rifampicin and isoniazid (82). The WHO has recently recommended the use of Genoscholar PZA TB II for detecting resistance to pyrazinamide in tuberculosis and Genoscholar FQ+KM-TB II for detecting resistance to fluoroquinolone and kanamycin in tuberculosis, based on gene mutations (83).

LPA offers rapid and targeted detection of known resistance mutations with a simplified workflow, but it may miss novel mutations. WGS, on the other hand, provides comprehensive genomic data but requires more resources, time, and expertise. The choice between the two techniques depends on the specific diagnostic and research needs, available resources, and the prevalence of drug resistance mutations in the population of interest.

The Truenat MTB/TruPlus test is a molecular diagnostic assay. It is a rapid test designed for the detection of *Mtb* and the identification of rifampicin resistance in clinical specimens (84). This test is based on real-time PCR technology and is specifically designed for use in resource-limited settings, making it a valuable tool for diagnosing TB, including drug-resistant TB (Chaitali Nikam, 2013).

The Truenat MTB/TruPlus test has gained recognition for its accuracy, speed, and suitability for point-of-care applications. In 2019, the WHO recommended the Truenat MTB assay as a molecular diagnostic test for TB, including the detection of rifampicin resistance (84).

Several commercial real-time PCR tests are available for the detection of *Mtb*, offering high sensitivity and specificity. Abott RealTime MTB is a real-time PCR assay designed to detect *Mtb* in clinical specimens (85). It is a highly sensitive and specific test that can provide rapid results. Roche's Cobas TaqMan MTB test is another real-time PCR assay for the detection of *Mtb*. It is known for its accuracy and automation capabilities. The Roche LightCycler system is used for real-time PCR-based detection of *Mtb*, providing accurate and rapid results. The Amplicor MTB PCR test, previously available from Roche, was used for *Mtb* detection, but it has been replaced by newer assays in many settings. In addition to *Mtb* detection, several commercial real-time PCR tests are available for the detection of drug resistance mutations in *Mtb*. These tests are crucial for identifying resistance to key anti-TB drugs. Some examples include Cobas MTB RIF/INH that is designed to detect resistance to rifampicin (RIF) and isoniazid (INH), which are critical first-line anti-TB drugs (86). Abbott RealTime MTB RIF used to detect rifampicin resistance in *Mtb* (85). The FluoroType MTBDR assay is used for the simultaneous detection of *Mtb* and resistance to rifampicin and isoniazid. These commercial real-time PCR tests offer high sensitivity and specificity for *Mtb* detection and drug resistance testing. They play a crucial role in improving the accuracy of TB diagnosis and guiding appropriate treatment, especially in cases of drug-resistant TB.

## Composite reference standard

Delay in diagnosis of paucibacillary TB has affected the efforts taken by Control Programs to curb its spread. Interventions to control spread of TB require more accurate and rapid diagnostic tests (87). Several gold standards have been used to improve the accuracy of diagnosing extrapulmonary TB. CRS combines several diagnostic tests and clinical criteria to create a more comprehensive reference standard. The CRS typically includes a combination of culture, histopathology, clinical follow-up, and response to anti-TB treatment (87). For example, a patient might be diagnosed with extrapulmonary TB if they have clinical symptoms consistent with TB, a positive histopathology result showing granulomas or AFB, and a favorable clinical response to anti-TB treatment. The CRS takes into account the limitations of individual tests and aims to improve diagnostic accuracy by using a combination of methods. It is important to note that the choice of diagnostic method depends on the type of extrapulmonary TB suspected and the available resources. In cases where the bacillary load is very low, a combination of tests and clinical criteria is the most reliable approach to achieve an accurate diagnosis. Advances in molecular diagnostics, such as NAATs, have significantly improved the diagnosis of extrapulmonary TB, but a multidisciplinary approach often remains necessary to ensure accurate and timely diagnosis (87).

## Conclusions

Numerous techniques have been developed to quickly identify and perform DST on *Mycobacteria* isolates through direct detection and species identification. The use of molecular methods has significantly reduced turn-around-time for TB diagnosis from weeks to days. However, while some techniques are straightforward, others are technically complex and may increase the overall cost of TB diagnosis. The WHO has recognized and supported various new technologies that have the potential to greatly enhance the diagnosis and treatment of patients, particularly those with drug-resistant TB. Although many novel technologies have been endorsed by the WHO, they may not be accessible to resource-limited countries, with high TB burden due to their cost and technical requirements. Therefore, conventional diagnostic methods such as culture and DST may still be necessary, and the new technology may not completely replace them.

Conventional culture-based diagnosis is still essential for detecting TB in patients who are smear-negative, and traditional DST is necessary to guide appropriate treatment choices. While molecular detection of resistance is reliant on identifying the specific mutation associated with drug resistance, other mechanisms of resistance may develop, or new mutations may arise that the test is not designed to detect.

Timely and accurate diagnosis of TB is essential for effective treatment, monitoring and disease control. The GeneXpert MTB/RIF Assay offers rapid, point-of-care diagnosis with simultaneous resistance detection, making it a cornerstone in TB management and could therefore become a game changer. Different diagnostic tests and assays have various strengths and weaknesses and the “best” test vary depending on the specific context, resources, and objectives. However, the GeneXpert MTB/RIF Assay is a leading option for TB diagnosis due to its speed and simultaneous resistance detection. Line Probe Assays, particularly the Hain GenoType MTBDRplus, are also valuable for their comprehensive drug resistance profiling. Whole Genome Sequencing is the gold standard for in-depth genetic analysis but not practical for routine clinical use due to cost and equipment requirements. The “second best” option would depend on whether rapid point-of-care or broader genetic insights are prioritized, such as LAMP for faster results, or WGS for research and surveillance. Therefore, a combination of diagnostic tests is needed to ensure accurate and timely results for TB diagnosis and patient management.

## Author contributions

NM: Conceptualization, Formal analysis, Project administration, Supervision, Visualization, Writing–original draft, Writing–review & editing. NS: Conceptualization, Writing–original draft. JB: Conceptualization, Data curation, Validation, Writing–original draft, Writing–review & editing. FB: Conceptualization, Data curation, Investigation, Methodology, Supervision, Visualization, Writing–original draft, Writing–review & editing. MI: Formal analysis, Writing–review & editing. AA: Conceptualization, Formal analysis, Supervision, Writing–review & editing. PB-K: Formal analysis, Methodology, Supervision, Validation, Visualization, Conceptualization, Writing–review & editing.
